# The Phenomenon of Neutrophil Extracellular Traps in Vascular Diseases

**DOI:** 10.1007/s00005-018-0505-y

**Published:** 2018-02-05

**Authors:** Dorota Dąbrowska, Ewa Jabłońska, Marzena Garley, Jolanta Sawicka-Powierza, Karolina Nowak

**Affiliations:** 1 Department of Immunology, Medical University of Bialystok, J. Waszyngtona 15A, 15-269 Bialystok, Poland; 2 Department of Family Medicine, Medical University of Bialystok, Mieszka I 4B, 15-054, Bialystok, Poland

**Keywords:** Neutrophils, NETs, Atherosclerosis, Thrombosis, Small-vessel vasculitis, AAV

## Abstract

Vascular diseases constitute a global health issue due to the increasing number of cases of patients with these diseases. The pathogenesis of the majority of these diseases, including atherosclerosis and thrombosis, is complex and not yet fully understood. One of the major causes for their occurrence can be immune disorders resulting in the development of a chronic inflammation within the vessels. In recent years, studies have placed emphasis on the role of neutrophils in the development of these diseases, i.e., the discovery of neutrophil extracellular traps (NETs) demonstrated that the structures released by the cells may contribute to the enhancement of inflammatory reactions and cell damage. This article summarizes current knowledge on the role of NETs during atherosclerosis, thrombosis and small-vessel vasculitis, especially in antineutrophil cytoplasmic antibody (ANCA)-associated small-vessel vasculitis (AAV).

## Introduction

Vascular diseases constitute a serious clinical, social and economic issue in the modern world. For many years, they have remained the major cause of mortality in developed countries. In recent years, studies have demonstrated that both innate and adaptive mechanisms of immune response are the basis for the majority of vascular diseases, including atherosclerosis and thrombosis (Chamorro and Hallenbeck 2006). Recently, interest in neutrophils has increased, as, according to reports, they play a significant role in the development and progression of cardiovascular diseases (Ionita et al. 2010; von Brühl et al. 2012). One of the major reasons for the great number of experiments conducted on these immunocompetent cells was the recent discovery of one of their functions—the formation and release of neutrophil extracellular traps (NETs). The literature reports that the excessive production of the NETs, stemming from the disturbed balance between the production and destruction of NETs, may contribute to the damage of vascular endothelium and to uncontrolled formation of thrombus in their lumina. The available literature data show that NETs may be involved in the development of atherosclerosis and thrombosis and also in small-vessel vasculitis (SVV) (Borissoff et al. 2013; Fuchs et al. 2010; Kessenbrock et al. 2009; Warnatsch et al. 2015).

### Neutrophil Extracellular Traps

Neutrophils represent the most abundant (50–75% of the total number of the peripheral blood leukocytes) population of white blood system cells. Neutrophils are one of the first effector cells recruited at the inflammation site, where they constitute the first line of defense in the combat against a wide range of microorganisms. Moreover, neutrophils can affect the recruitment and activation of other immunocompetent cells, as well as participate in regenerative processes of tissues. For many years, the antipathogenic activity of neutrophils was linked solely to the elimination of pathogens through phagocytosis, including degranulation of antibacterial proteins and generation of reactive oxygen species (ROS) (Borregaard 2010; Mantovani et al. 2011).

A breakthrough in the study on these cells came from the results of the study of Brinkmann et al. (2004) published in *Science*. The authors of this pioneering article recently detected an additional property of neutrophils consisting of the generation of NETs (Brinkmann et al. 2004). Thanks to this discovery, scientists began to realize that the earlier information on neutrophils was insufficient and a new analysis of their functions should be performed. Brinkmann et al. (2004) indicated that the NET structure consists of clusters of thin fibers and globular domains able to aggregate and form larger strands of traps. These scientists also revealed that NETs may be subjected to degradation by DNase but not proteases, which confirmed that the deoxyribonucleic acid (DNA) is one of the main NET elements (Brinkmann et al. 2004). Apart from DNA, NETs, contain numerous proteins originating from the primary neutrophil granules: myeloperoxidase (MPO), neutrophil elastase (NE), proteinase 3 (PR3); secondary neutrophil granules: lactoferrin, pentraxin 3 and tertiary neutrophil granules: gelatinase (Jaillon et al. 2007; Urban et al. 2009; Wartha et al. 2007). In the globular domains of NETs, histones: H1, H2A, H2B, H3, H4 are the dominant group of proteins and they constitute approx. 70% of all proteins related to NETs (Brinkmann et al. 2004; Dwivedi et al. 2014).

Formation and release of NETs demonstrates a unique form of cell death, known as NETosis, which cannot be compared to other known death programs, including apoptosis and necrosis (Fuchs et al. 2007). The proper course of NETosis requires the activity of the NADPH oxidase complex, histone citrullination, being the posttranslational modification of proteins, catalyzed by peptidylarginine deiminases (PADs), in particular by the PAD4 isoform and autophagy phenomena (Fuchs et al. 2007; Neeli et al. 2008; Remijsen et al. 2011).

Numerous studies have demonstrated that NETs play a crucial role in the body’s immune reaction to an infection. A release of these structures to the extracellular space enables the entrapment and elimination of many pathogens, including bacteria: *Salmonella typhimurium, Shigella flexneri* (Brinkmann et al. 2004), fungi: *Candida albicans* (Urban et al. 2006), *Aspergillus fumigatus* (McCormick et al. 2010), protozoans: *Toxoplasma gondii* (Abi Abdallah et al. 2012), *Leishmania amazonensis* (Guimarães-Costa et al. 2009) and viruses: human immunodeficiency virus 1 (HIV-1) (Saitoh et al. 2012), influenza A virus H1N1 (Narasaraju et al. 2011). In their review article, Kaplan and Radic (2012) described that by forming a physical barrier, NETs facilitate the degradation of bacterial and viral factors of virulence, and thereby prevent the spread of microorganisms.

But, despite these significant advantages resulting from the formation of NETs, numerous scientific studies report on the pathological role of these structures. As has been observed, the process of the generation and elimination of NETs should be strictly regulated. An excessive number of these structures formed in an inappropriate place and time may cause numerous undesirable and unfavorable changes in an organism (Manda et al. 2014). In the light of current knowledge, formation of NETs, carrying the nuclear material in the form of DNA and enzymes, such as MPO or NE, constitutes a potential developmental factor for autoimmunization and cardiovascular disorders.

### NETs and Atherosclerosis

Atherosclerosis, a civilization disease, has become one of the most common health problems in recent years. The development of atherosclerosis is caused by damaged endothelium, a chronic response of the vessel walls with an inflammatory character, resulting in adhesion of leukocytes and blood platelets and an increase in the permeability of vessels for lipid compounds, primarily low density fraction. Because of the accumulation of the immune system cells and lipids, atherosclerotic plaques are developed, surrounded by smooth muscle cells (Hansson 2005). The atherosclerotic process is influenced by a series of states, such as obesity, hypertension, diabetes, dyslipidemia, which increase the risk of a rapid progression of atherosclerotic changes leading to the occurrence of significant disorders in the function of vital organs (Scott 2002; Singh et al. 2002). The available literature data show that atherosclerosis should be treated not only as a disease related to lipid disorders, but also as a chronic inflammatory disease, for instance due to the cells of the immune system found in atherosclerotic plaques, including T lymphocytes, macrophages, granulocytes, which, by releasing inflammatory mediators (cytokines, growth factors), influence the development of these atherosclerotic plaques (Falk 2006; Jawień 2008; Singh et al. 2002; Weber et al. 2008).

Initially overshadowed, neutrophils gained more importance when it turned out that they can occur in different regions of an atherosclerotic plaque, including in the fibrous cap, in the shoulder, and in areas toward the media (also known as the base of the plaque). As described, neutrophils play a significant role in both atherosclerosis pathogenesis as well as in the destabilization of atherosclerotic plaque (Ionita et al. 2010). Thanks to their capacity to form numerous factors, including ROS and cytokines, neutrophils participate in the promotion of systemic inflammatory reactions and influence the local concentration of different immunocompetent cells modulating the permeability of endothelial cells (Baetta and Corsini 2010). In their review article, Chistiakov et al. (2015) described that for a chronic inflammation accompanying atherosclerosis, the activity of neutrophils may be directed at their own cells and also contribute to gradual vessel damage.

An additional stimulus for further study on the role of neutrophils during the atherosclerotic process was the discovery of NETs. Megens et al. (2012) were among the first to detect NET formation in a mouse atherosclerosis model as well as in patients who were subjected to the procedure of endarterectomy, i.e., removal of atherosclerotic plaques. The study conducted by Knight et al. (2014) further proved that neutrophils isolated from mice with atherosclerosis are more susceptible to NET formation. Moreover, the authors of this publication demonstrated that inhibition of PAD4 by Cl-amidine results not only in the reduction of NET formation, but also protects against the development of atherosclerosis and arterial thrombosis, which suggests their significant role in the pathogenesis of these diseases (Knight et al. 2014).

**Fig. 1:**
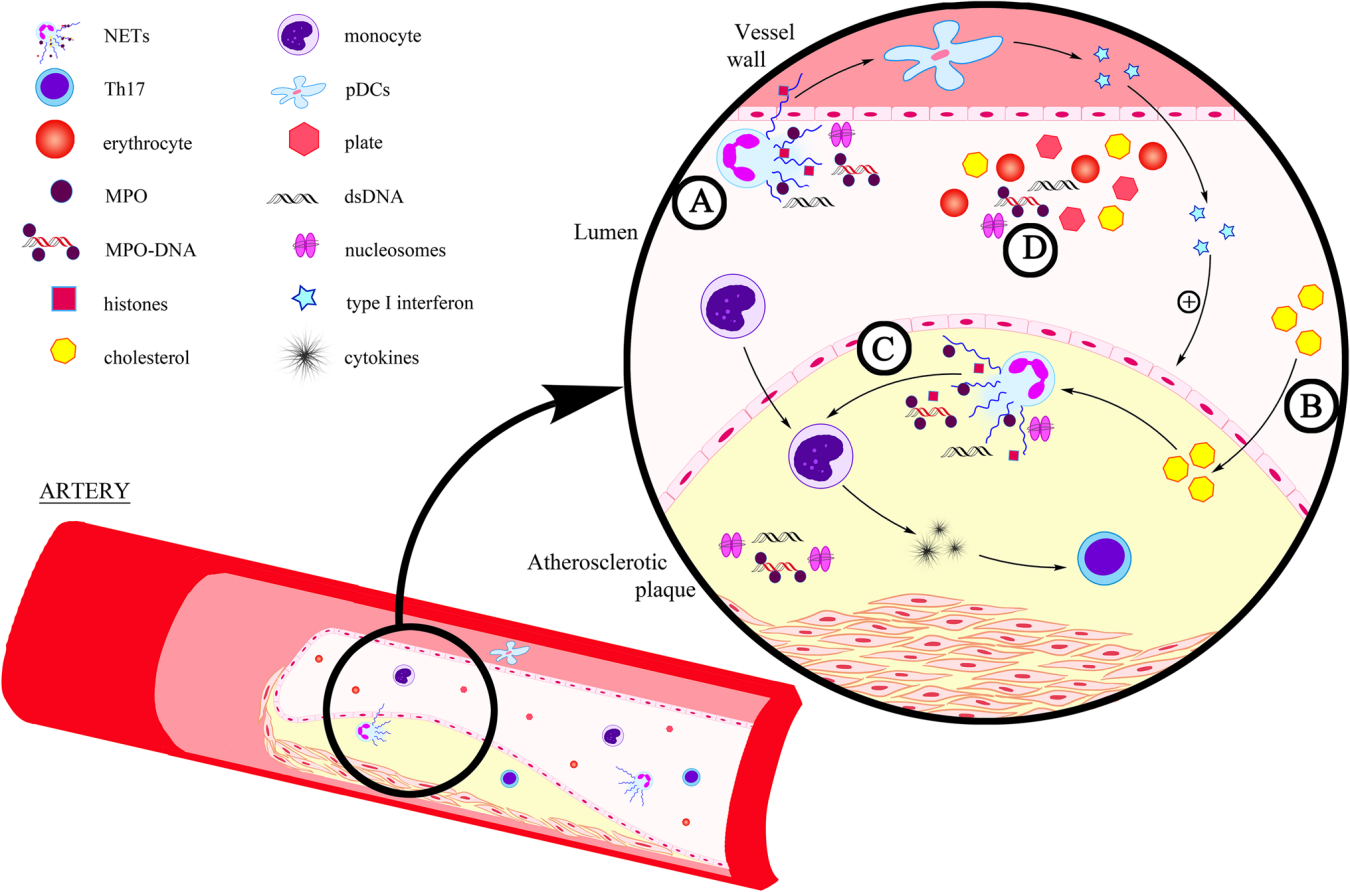
NET formation in atherosclerosis. **a** NETs may influence activation of plasmacytoid dendritic cells (pDC), releasing large amounts of type I interferon facilitating the development of atherosclerotic changes (Döring et al. 2012); **b** Cholesterol crystal induce NETosis (Warnatsch et al. 2015); **c** NETs stimulate macrophages to release cytokines, activating Th17 lymphocytes, which enhance the recruitment of immunity cells in atherosclerotic plaques (Warnatsch et al. 2015); **d** NET markers circulating in the bloodstream (nucleosomes, double-stranded DNA (dsDNA) and MPO-DNA) (Borissoff et al. 2013)

Borissoff et al. (2013), who aimed to determine the relationship between NET release and coronary atherosclerosis and the presence of prothrombotic state, revealed that elevated levels of NET markers—double-stranded DNA, nucleosomes and MPO-DNA complexes were significantly correlated with the occurrence of serious cardiovascular events. According to these scientists, these biomarkers may prove useful in the forecasting of coronary disease (Borissoff et al. 2013). Interesting observations were published by Warnatsch et al. (2015) who, using a mouse model of atherosclerosis, demonstrated that cholesterol crystals influence the induction of NETosis. They observed that NETs constituted large amorphic structures within the atherosclerotic changes in mice which had previously been introduced with a high-lipid diet to induce hypercholesterolemia. Moreover, based on the results of their study, these scientists suggested that NETs may also contribute to the development of atherosclerosis through modulation of cytokine formation. Evidence for this assumption was the finding of elevated levels of interleukin (IL)-1α, IL-1β and IL-6 in the blood plasma of mice with excessive generation of NETs (Warnatsch et al. 2015) (Fig. 1).

Literature data demonstrate that NETs constitute a connection between innate and adaptive immune response and may influence the acuteness of atherosclerosis, i.e., through activation of T lymphocytes and antigen-presenting cells. This is demonstrated in the study conducted by Tillack et al. (2012), which revealed that NETs have the ability to directly prime T lymphocytes by decreasing their activation threshold and thereby contributing to an enhanced adaptive immune response. Moreover, the results of the study obtained by Warnatsch et al. (2015) showed that NETs may stimulate macrophages to release cytokines, activating Th17 lymphocytes, which enhance the recruitment of immune cells in atherosclerotic plaques and which are thus capable of influencing the development of inflammation within vessels. According to other experiments, NETs may also influence activation of plasmacytoid dendritic cells identified in murine and human atherosclerotic plaques, releasing large amounts of type I interferon facilitating the development of atherosclerotic changes (Döring et al. 2012).

Oxidative stress, defined as the balance disorder between the formation of ROS and the oxidative capacity of an organism, constitutes the integral part of the pathogenesis of atherosclerosis (Madamanchi and Runge 2013). Recent results of the study conducted by Wang et al. (2017) indicated a significant relationship between mitochondrial oxidative stress (mitoOS) and generation of NETs in aged mice with atherosclerosis. Authors of this publication observed that inhibition of mitoOS in aged mice leads to reduction of NET formation, which could not be observed in younger specimens. These results uncover a new possibility for prevention of atherosclerosis development during aging.

### NETs and Thrombosis

Thrombosis is one of the most serious vascular diseases and it consists of the formation of a thrombus in the lumen of blood vessels. Following the hypothesis formulated in 1856 by Rudolf Virchow, the main stimuli contributing to the development of thrombosis are: decreased blood flow, damage to the vessel walls and hypercoagulation (Bagot and Arya 2008). Numerous factors increasing the risk of this disease are known, including: obesity, long-lasting immobilization, tumors, infections, injuries and surgical procedures. Susceptibility to thrombosis may also stem from congenital or acquired coagulation disorders (Cushman 2007).

Many literature reports indicate the existence of a relationship between blood coagulation and mechanisms of the innate immune response of an organism. Activated thrombocytes exhibit the capacity to bind a large number of leukocytes, including neutrophils, and to generate leukocyte—platelet aggregates (Zarbock et al. 2007). Furthermore, neutrophils may play a significant role in the initial stage of fibrin formation and clot formation resulting in their interaction with endothelium cells damaged by LFA-1/ICAM-1 adhesion molecules (Darbousset et al. 2012). Researchers have also noticed that generation of NETs may influence the thrombogenicity of these cells during sepsis and deep vein thrombosis (DVT) (Kambas et al. 2012; von Brühl et al. 2012).

Numerous publications provide evidence that NETs possess prothrombotic properties and may function as a scaffolding for thrombocytes and erythrocytes morphotic elements of the blood, which including fibrin participate in the formation of a clot (Brill et al. 2012; Fuchs et al. 2010). An important step on the way to an understanding of the role of NETs in thrombosis was the study of Fuchs et al. (2010), who observed that a thrombus originating from a baboon experimental model of DVT contains components of NETs, including extracellular DNA, histones H3 and a complex DNA/H2A/H2B. The authors of the publication also revealed that, under in vivo conditions, NETs occur in co-location with the fibers of fibrin. From their observations, they concluded that the potential strict cooperation of NETs and fibrin may influence the organization and stability of a clot (Fuchs et al. 2010). These reports have been confirmed by Varjú et al. (2015) who demonstrated that histones and DNA increase the diameter of fibrin fibers, which constitute the scaffolding for blood clots under in vitro conditions. Furthermore, these scientists observed that DNA influences a decrease in the activity of plasminogen, while histones contribute to the inhibition of thrombin effect through antithrombin.

**Fig. 2:**
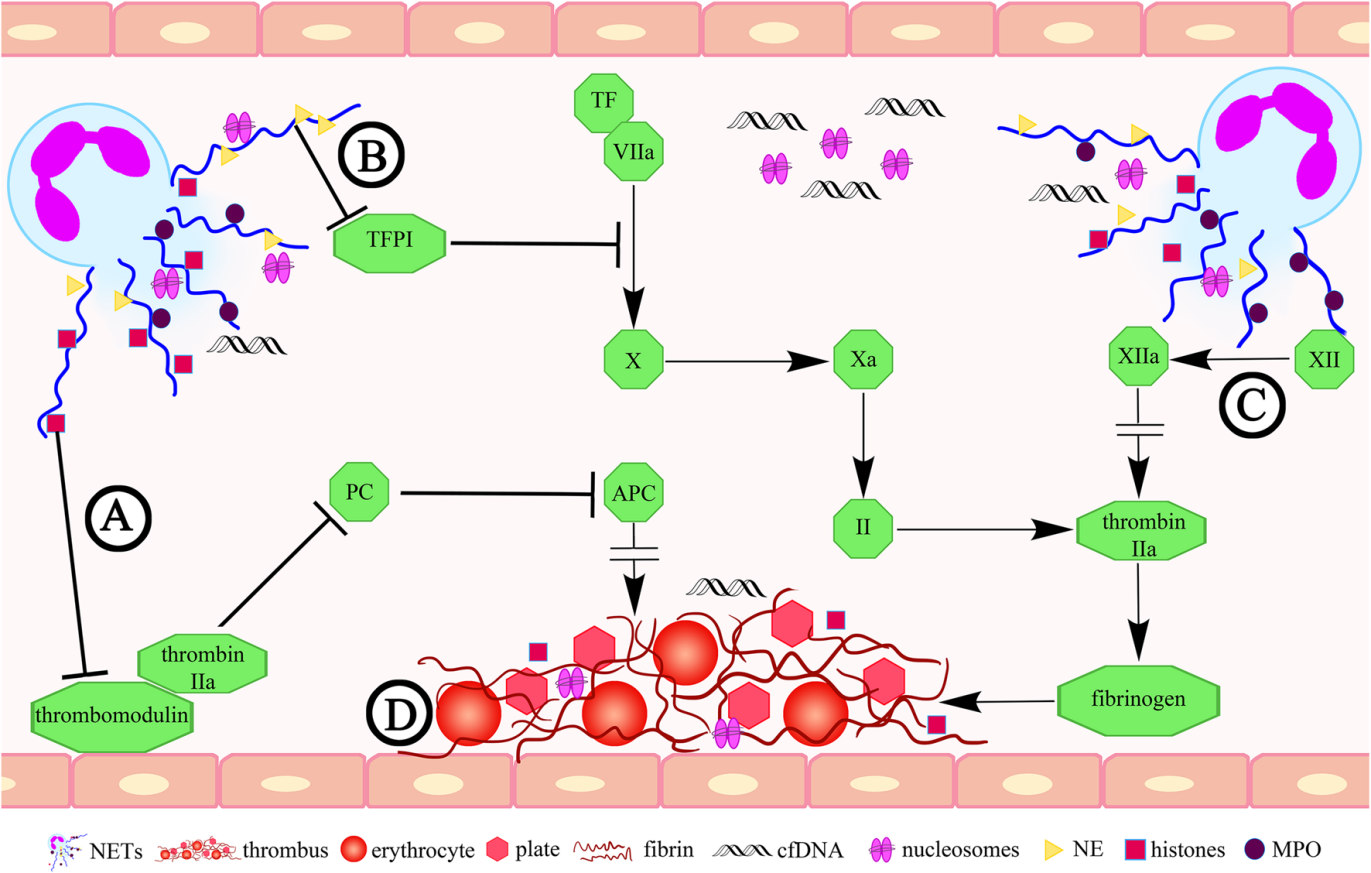
The role of NETs in thrombosis. **a** Histones related to a NET may affect the inhibition of the protein C activation by influencing the thrombomodulin (Ammollo et al. 2011); **b** NET-derived elastase may impact on the tissue factor pathway inhibitor (TFPI) inactivation (Massberg et al. 2010); **c** NETs may influence the development of thrombosis through binding and activation of the blood coagulation factor XII (von Brühl et al. 2012); **d** NETs play a role of scaffolding for thrombocytes and erythrocytes (Brill et al. 2012; Fuchs et al. 2010). *APC* activated protein C, *TF* tissue factor, *NE* neutrophil elastase, *PC* protein C, *cfDNA* cell-free DNA, *MPO* myeloperoxidase

Furthermore, the results of the study conducted by von Brühl et al. (2012) on a mice DVT model demonstrated that NETs formed and released by neutrophils may influence the development of DVT through binding and activation of blood coagulation factor XII. As reported in the scientific literature, during arterial thrombosis, procoagulant activity may also be enhanced as a result of an indispensable element of NETs neutrophil elastase, which influences inactivation of the tissue factor pathway inhibitor (TFPI) (Massberg et al. 2010). But, this assumption was not confirmed for the experimental DVT model. Martinod et al. (2016) revealed that a deficiency of NE does not contribute to inhibition of the NETs generation, which may indicate that the NE-dependent TFPI inactivation is not of significance in the DVT pathogenesis (Fig. 2).

Fuchs et al. (2010), in their experimental study, observed a completely new mechanism of antithrombotic action of heparin. This well-known anticoagulant may influence destabilization of NETs by releasing histone proteins from a nucleoprotein complex, thus resulting in a restriction of the formation of these structures in DVT under in vivo conditions (Fuchs et al. 2010). The study by Martinod et al. (2013) indicated that suppression of thrombus formation is also possible in PAD4-knockout mice (which is indispensable for NETs generation).

Of special interest seems to be the study conducted by Brill et al. (2012), which enabled the discovery of the possibility to prevent thrombus formation with the contribution of DNase I, which, as known, disintegrates NETs, and thus can prevent the initiation of blood coagulation cascade resulting in thrombosis. The observations of von Brühl et al. (2012) published several months later also demonstrated that DNase I influences the restriction of NET generation and inhibits the development of DVT in mice. The recent study of Jiménez-Alcázar et al. (2015) demonstrated that a low level of DNase I leads to an inefficient removal of excessive amounts of NETs, contributing to the promotion of thrombosis in patients with thrombotic microangiopathy.

The protein C has a significant role in the inhibition of blood coagulation, thus preventing thrombus formation. Originally, the protein C is released in the inactive form, then it is transformed into active form under the influence of the thrombin and thrombomodulin interaction (Esmon 2003). Ammollo et al. (2011) indicated that an excess of extracellular histones related to NETs may influence the inhibition of the protein C activation by influencing the activity of the endothelial cofactor of this glycoprotein—thrombomodulin, resulting in an elevated generation of thrombin, which participates in the transformation of fibrinogen into insoluble fibrin, and thus to the formation of thrombus. The prothrombotic role of histones is also reported in the study conducted by Semeraro et al. (2011), who observed that these proteins may lead to the induction of the blood coagulation cascade with formation of thrombin thanks to binding and activation of thrombocytes through Toll-like receptor 2 (TLR2) and TLR4.

The initial experiments on the involvement of NETs in the development of thrombosis were conducted on animal experimental models. Van Montfoort et al. (2013) were among the first to assess the role of NETs in patients with DVT. The results of their study demonstrated that an elevated level of circulating nucleosomes and NE–α1-antitrypsin complexes were linked to a three-fold higher risk of DVT. Moreover, the observations of Diaz et al. (2013) revealed that the blood plasma of patients with an acute form of DVT has a significantly elevated level of cell-free DNA (cfDNA), which is a potential marker of NET formation. The scientists also observed a strong positive correlation between the cfDNA level and certain markers of DVT, including the C reactive protein, D-dimers, MPO, von Willebrand factor and a negative correlation with the ADAMTS13 metalloprotease (Diaz et al. 2013). Furthermore, the study conducted by Savchenko et al. (2014) showed that in patients with venous thromboembolism, the NETs participate in the formation of venous thrombosis, thus indicating the significant role of these structures in the progression of the disease.

### NETs and Small-Vessel Vasculitis

Small-vessel vasculitis is a systemic autoimmune disease. In its course, an inflammation and necrosis occur in the walls of small vessels including arterioles, venules and capillaries. As reported in the literature, one cannot exclude the possibility that the process also includes mid-sized arteries and veins (Jennette and Falk 1997).

According to the classification established in 2012 at the International Chapel Hill Consensus Conference Nomenclature of Vasculitides (Jennette 2013), SVV are divided into:

- antineutrophil cytoplasmic antibody (ANCA)-associated vasculitides (AAV), which are further divided into: microscopic polyangiitis (MPA), granulomatosis with polyangiitis (GPA) and eosinophilic GPA (EGPA);
- immune-complex associated vasculitides, including: anti-glomerular basement membrane disease, cryoglobulinemic vasculitis, IgA vasculitis (Henoch-Schönlein) and hypocomplementemic urticarial vasculitis (anti-C1q vasculitis) (Jennette 2013).

The AAV pathogenesis is complex and it is influenced by both genetic predispositions as well as environmental factors (environmental pollution, drugs, infections), leading to induction and excessive activation of lymphocytes and autoantibodies, contributing to tissue damage and predisposal to disease recrudescence (Cartin-Ceba et al. 2012). Numerous experimental studies and clinical observations have confirmed the pathogenetic role of ANCA, mostly myeloperoxidase (MPO-ANCA) and proteinase 3 (PR3-ANCA) (Falk et al. 1990; Schreiber et al. 2004). Literature data indicates that PR3-ANCA is most common in GPA patients, whereas MPO-ANCA occurs more frequently in patients with MPA (Jennette and Falk 1998; Rowaiye et al. 2015).

Following the discovery of NETs, the potential relationship was observed between formation of these structures and ANCA-associated vasculitis progression. This is confirmed by the presence of the autoantigens (MPO and PR3) in the NETs. Kessenbrock et al. (2009) noticed that anti-PR3-antibodies stimulate neutrophils to form and release NETs in the course of AAV. Moreover, these scientists demonstrated that in the majority of patients with AAV who have complications in the form of glomerulonephritis, NETs are accumulated within the structural elements of the organ, suggesting that the NETs may contribute to the progression of AAV (Kessenbrock et al. 2009). Several months later, a description was published in the scientific literature of a case of a patient with SVV in which in skin biopsy material using immunofluorescence, anti-MPO antibodies and NETs were detected (Abreu-Velez et al. 2009).

**Fig. 3:**
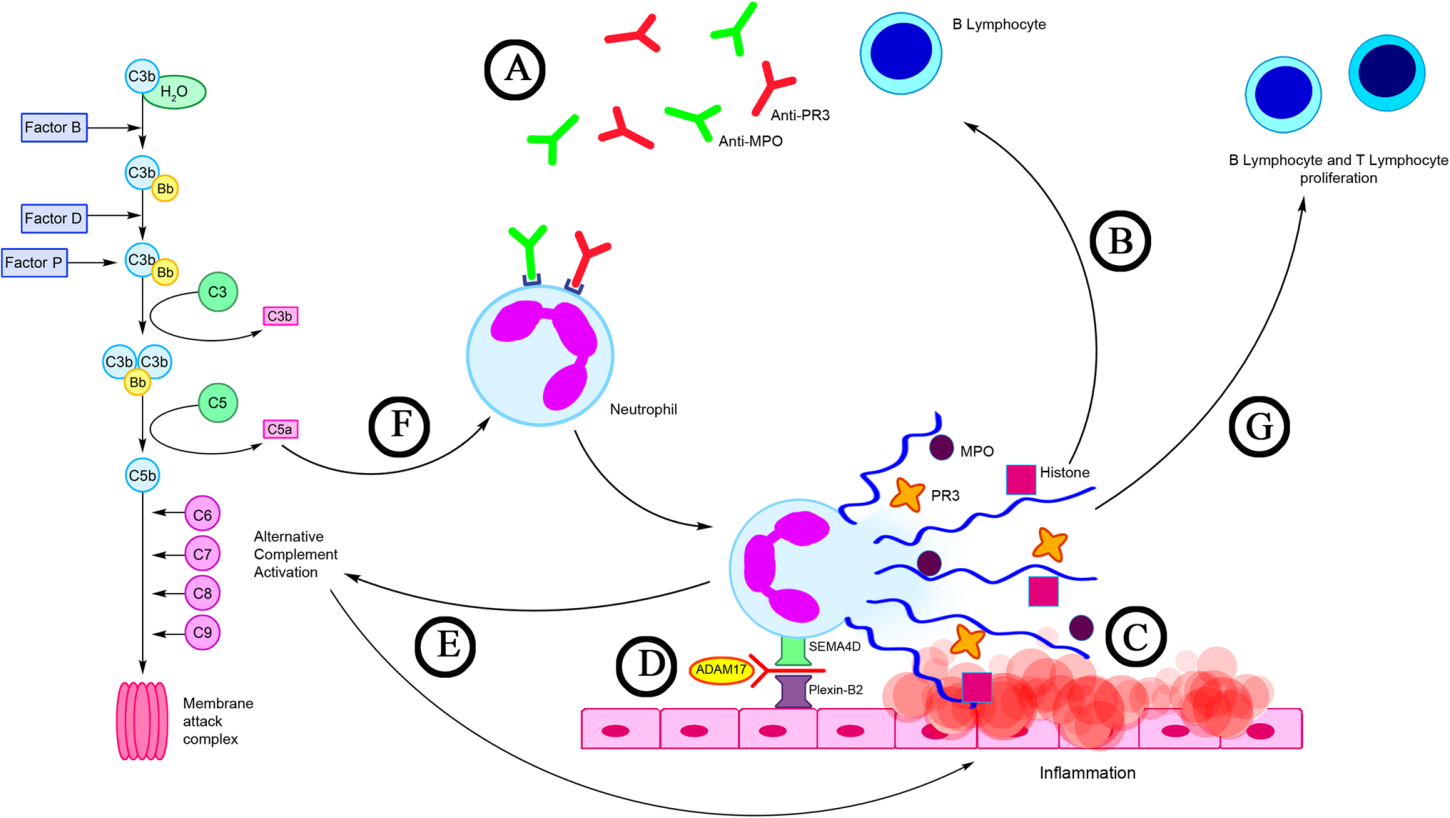
NETs in AAV. **a** ANCA antibodies (e.g., anti-MPO and anti-PR3) stimulate neutrophils to form and release NETs (Kessenbrock et al. 2009); **b** NETs are a source of autoantigens (MPO, PR3) and a factor supporting the production of ANCA antibodies by autoreactive B lymphocytes (Sangaletti et al. 2012); **c** Long-term exposure to NETs components (MPO, histones, PR3) may contribute to direct damage to endothelial cells; **d** Proteolytic cleavage of semaphorin 4D (SEMA4D) with ADAM metallopeptidase domain 17 (ADAM17) involvement, which is observed in patients with AAV, may have an effect on the severity of NET generation (Nishide et al. 2017); **e** NETs may activate an alternative complement pathway that plays an important role in enhancing inflammation process in AAV (Wang et al. 2015); **f** C5a is a strong chemoattractant and activator of neutrophils (Chen et al. 2017); **g** NETs are the link between innate and adaptive immunity. NETs may affect B lymphocyte and T lymphocyte proliferation (Lange et al. 2017)

Propylthiouracil (PTU) is a drug with a proven impact on the development of AAV, which has been found to contribute to the induction of ANCA. Due to its thyrostatic action, PTU is administered to treat hyperthyroidism (Cao and Lin 2013). Recent reports have revealed that PTU can affect the NET generation. Interesting observations were obtained by Nakazawa et al. (2012a) who indicated that PTU may influence a change of NET conformation released by human neutrophils induced by PMA both in vitro and in vivo. These scientists observed that a change of the spatial layout of the NETs leads to their impaired degradation with the contribution of DNase I. An explanation of the occurrence of this phenomenon is the fact that PTU metabolites impede identification of the cutting sites of the endonuclease through binding NETs to the DNA. According to these researchers, this may constitute the critical aspect in the pathogenesis of AAV (Nakazawa et al. 2012a). A continuation of the study conducted by Nakazawa et al. (2014) supports earlier reports by Kessenbrock et al. (2009), concerning the strong ability of ANCA for induction of NETosis in patients with AAV. Furthermore, these authors discovered that NETs degradation in patients with AAV is significantly inhibited by the low level of DNase I activity and presence of anti-NET antibodies (Nakazawa et al. 2014). In contrast Sangaletti et al. (2012) demonstrated that NETs are highly immunogenic and that they may lead to the induction and support of autoimmunization. They observed that myeloid dendritic cells (mDCs) may capture some NET components, i.e., MPO and PR3, and then present them as antigens. Furthermore, the authors revealed that in skin samples obtained from patients with MPA, mDCs were in direct contact with NETs (Sangaletti et al. 2012) (Fig. 3).

The results of Söderberg et al. (2015) showed that in the circulation of patients with AAV an elevated level of NET remnants is found, which has a positive correlation with the activity of the disease and is negatively correlated to the number of ANCA antibodies in the remission period of the disease. The authors also observed that the neutrophils of patients with AAV exhibit a stronger tendency for spontaneous cell death through NETosis (Söderberg et al. 2015). On the other hand, Lange et al. (2017) have revealed in their latest publication that in the serum of patients with GPA, cfDNA concentration is significantly increased, which may indicate significant participation of these structures in the development of this disease. They also observed that NETs may influence the B lymphocyte maturation and T lymphocyte (CD4^+^) and B lymphocyte (CD19^+^) proliferation (Lange et al. 2017).

Of special interest appears the study conducted by Natorska et al. (2017), who were among the first to describe that neutrophils of patients with EGPA are capable of NETs generation. Authors of this publication further reported on the participation of eosinophils in the induction of NETosis, as they observed, the number of these cells correlated with the number of NETs in all examined EGPA patients.

It has been known for a long time that thromboembolic complications accompany vasculitis (Tomasson et al. 2009). The frequency of their incidence increases along with the activity of the disease, which may be linked with the exacerbation of the inflammation covering the venules (Stassen et al. 2008). The search for the exact reasons of the development of these complications allowed researchers to notice the share of pro-inflammatory NETs, which constitute a significant aspect both in the pathogenesis of thrombosis as well as AAV. The study of Nakazawa et al. (2012b) demonstrated that the thrombus collected post mortem from a female patient with MPA contained a large amount of NETs. Kambas et al. (2014) indicated that the expression of the tissue factor present in NETs and in microparticles released by neutrophils may be of importance in the induction of thrombosis in patients with AAV.

## Conclusion

The literature reports cited in this study show that the recently discovered function of neutrophils, which is the formation of NETs, plays an important role in the development of vascular diseases, including atherosclerosis, thrombosis and SVV, which is confirmed by the elevated level of NET markers, including cfDNA and nucleosomes. According to numerous authors, an excessive amount of NETs formed within vessels may constitute a significant cause of chronic inflammation, which is one of the basic elements in the pathogenesis of these diseases. Therefore, inhibition of the generation of these structures may result in numerous favorable clinical effects for patients with vascular diseases. As has been hitherto observed, DNase I and the PAD4 inhibitor—Cl-amidine may influence the restriction of NET formation, thus protecting against the development of atherosclerosis and thrombosis however, these observations have been conducted so far only in animal experimental models.

Numerous aspects of neutrophil hyperactivity and their function in vascular diseases are not entirely understood, thus it seems warranted to undertake studies aimed at the determination of the precise role of these cells, including the function of NETs in the pathogenesis of vascular diseases.

Enhancement of knowledge of the phenomenon of NET formation, creating a physical barrier in vessels, could lead to the development of new treatment strategies and improvement of the quality of life of patients suffering from these diseases in the future.
